# Roles for the coactivators CBP and p300 and the APC/C E3 ubiquitin ligase in E1A-dependent cell transformation

**DOI:** 10.1038/sj.bjc.6603304

**Published:** 2006-08-01

**Authors:** A S Turnell, J S Mymryk

**Affiliations:** 1 Cancer Research UK Institute for Cancer Studies, The Medical School, The University of Birmingham, Edgbaston, Birmingham B15 2TT, UK; 2 Departments of Oncology and Microbiology & Immunology, University of Western Ontario, London, Ontario, Canada N6A 4L6

**Keywords:** *E1A*, adenovirus, transcription, cell-cycle, transformation, cancer

## Abstract

Adenovirus early region 1A (*E1A*) possesses potent transforming activity when expressed in concert with activated *ras* or *E1B* genes in *in vitro* tissue culture systems such as embryonic human retinal neuroepithelial cells or embryonic rodent epithelial and fibroblast cells. Early region 1A has thus been used extensively and very effectively as a tool to determine the molecular mechanisms that underlie the basis of cellular transformation. In this regard, roles for the E1A-binding proteins pRb, p107, p130, cyclic AMP response element-binding protein (CBP)/p300, p400, TRRAP and CtBP in cellular transformation have been established. However, the mechanisms by which E1A promotes transformation through interaction with these partner proteins are not fully delineated. In this review, we focus on recent advances in our understanding of CBP/p300 function, particularly with regard to its relationship to the anaphase-promoting complex/cyclosome E3 ubiquitin ligase, which has recently been shown to interact and affect the activity of CBP/p300 through interaction domains that are evolutionarily conserved in E1A.

## ADENOVIRUS AS A TOOL FOR DISSECTING THE MOLECULAR MECHANISMS OF CELLULAR TRANSFORMATION

The adenoviruses are a family of small nonenveloped viruses with linear double-stranded DNA genomes of about 35 kbp. They infect most animal species and cause a range of clinical diseases in humans. Fifty-one human adenovirus (HAdV) serotypes have been isolated to date and divided into six subgroups, termed A–F, according to a variety of criteria (Benko *et al*, 2000). Forty years ago, HAdV-12 was shown to induce tumours when injected into newborn hamsters, providing the first evidence that a human virus could be oncogenic (Trentin *et al*, 1962). Although only subgroup A HAdVs, such as HAdV-12, efficiently induce tumours in rodents, all HAdVs appear capable of cellular transformation in *in vitro* tissue culture systems. The differential oncogenicity observed in animals is attributed to the ability of the more oncogenic viruses to evade the host immune response, as cells transformed by otherwise nononcogenic serotypes will induce tumours in immunocompromised animals (Gallimore and Turnell, 2001).

**Figure 1:**
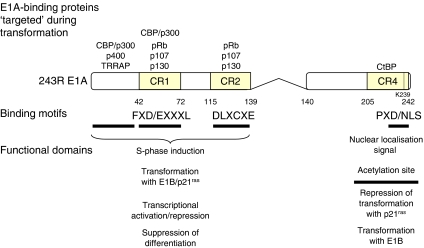
Linear depiction of the HAdV-5 243R E1A protein. The regions conserved between serotypes are labelled as CR1, CR2 and CR4; amino-acid ordinates depict CR boundaries. The domains required for the indicated E1A functions are indicated as black bars beneath the map. The location and consensus sequences of binding motifs necessary for interaction with CBP/p300, pRb and CtBP are indicated, as are the general regions of E1A implicated in binding to the specified cellular proteins. K239, the major acetylation site targeted by CBP/p300, is also depicted.

Expression of the leftmost adenoviral gene, termed early region 1A (*E1A*) is crucial for transformation and has been most extensively characterised in HAdV-5. The HAdV-5 *E1A* gene encodes two major proteins of 289 and 243 residues, which arise from differential splicing of the same transcript and differ only by the presence of an internal sequence of 46 amino acids in the larger protein (Figure 1). Sequence comparisons of the largest E1A proteins of several adenovirus serotypes identified four regions of sequence conservation, designated conserved regions (CR) 1, 2, 3 and 4 (Avvakumov *et al*, 2002), which are important for many of E1A's biological activities (see below).

In infected human cells, *E1A* is essential for a productive viral infection, although this can be bypassed using higher multiplicities of infection (Berk, 2005). The effects of E1A can be considered to be largely, if not completely, mediated by changes in transcription. Early region 1A is the first viral gene expressed after infection and is responsible for activating viral gene transcription. It also reprogrammes host cell gene expression, forcing quiescent cells to enter and pass through the cell cycle, and moreover, blocks cell differentiation (Gallimore and Turnell, 2001; Frisch and Mymryk, 2002; Berk, 2005). HAdV infection of human cells usually results in the death of the host cell and the release of progeny virus. Cell death precludes the possibility of malignancies resulting from human infection. For this reason, and probably others, adenoviruses are not generally thought to be a cause of human cancer. In contrast to human cells, HAdV infection of rodent cells is nonproductive. In this context, infection does not result in cell death and *E1A's* oncogenic properties become readily apparent. *Early region 1A* can efficiently immortalise rodent cells or fully transform them in cooperation with a second oncogene, such as the adenovirus *E1B* gene or activated *ras* (Gallimore and Turnell, 2001; Frisch and Mymryk, 2002; Berk, 2005). Despite its clear oncogenic properties, expression of *E1A* in previously transformed cells has shown that it can, in certain circumstances, also function as an antioncogene to suppress metastasis, angiogenesis and tumorigenicity *in vivo*, trigger apoptosis, and induce differentiation to an epithelial-like cell type (Mymryk, 1996; Gallimore and Turnell, 2001; Frisch and Mymryk, 2002).

## REQUIREMENT FOR CELLULAR E1A-BINDING PROTEINS IN THE TRANSFORMATION PROCESS

The E1A proteins act by directly binding to cellular regulators that play key roles in controlling gene expression and cell growth. Although neither the crystal nor solution structure is known for any of the E1A proteins, mutational analysis has clearly shown that the E1A proteins are organised as a collection of independent protein-binding modules that confer interaction with a number of cellular proteins (Gallimore and Turnell, 2001; Frisch and Mymryk, 2002; Berk, 2005). For example, E1A interacts with the cellular transcriptional corepressor CtBP via the short motif P*x*D/NLS (where *x* is variable) found in CR4 and binds the transcriptional repressor and cell-cycle regulator pRb through the core motif DL*x*C*x*E found in CR2. Moreover, E1A interaction with the cyclic AMP response element-binding protein (CBP)/p300 acetyltransferases requires the motif F*x*D/E*xxx*L found in CR1. Each of these sequences was originally identified within E1A, but has since been found in numerous cellular proteins that also interact with these targets. Presumably, these interaction motifs have been hijacked by *E1A* from cellular genes via recombination events, or the *E1A* gene has evolved independently, such that the protein mimics cellular protein interaction surfaces in order to provide selected functional advantages to E1A, and consequently the virus.

Given these properties, E1A has been instrumental in the identification of key cellular regulatory proteins involved in transformation. The most notable of these is the tumour suppressor pRb, which was the first E1A-binding protein to be identified. Indeed, the identification of pRb as an E1A-binding protein was the first demonstration of a physical link between an oncogene and a tumour suppressor gene (Whyte *et al*, 1988). Functionally, E1A binding to pRb activates host cell DNA synthesis in baby rat kidney (BRK) cells through promoting the release of the transcription factor E2F from pRb to activate transcription of the viral *E2A* gene and a number of cellular S-phase-specific genes. Mutational analyses have indicated that interference with the cell-cycle regulatory functions of pRb is required for cellular transformation by E1A (Gallimore and Turnell, 2001; Frisch and Mymryk, 2002; Berk, 2005). The ability of E1A to bind and remodel p400- and TRRAP- containing complexes through its N-terminal region is also important in transformation (Gallimore and Turnell, 2001). The C-terminal CR4 domain of E1A also functions in the transformation process, suppressing E1A/*ras*-mediated transformation, while enhancing E1A/E1B-mediated transformation (Mymryk, 1996; Frisch and Mymryk, 2002). It has been postulated that E1A interaction with CtBP is crucial for these activities. The interaction of E1A with CtBP has also been shown to relieve repression of cellular genes and promote mesenchymal to epithelial transition (Grooteclaes and Frisch, 2000). The functions of other C-terminal binding proteins in the transformation process await further investigation.

## INTERACTION OF E1A WITH CBP AND P300

The p300 protein was first identified through its specific interaction with the N-terminal region and CR1 of E1A (Eckner *et al*, 1994). Following characterisation of the *p300* gene, it was subsequently determined that E1A also binds the highly-related CBP (Arany *et al*, 1995). Before the formal characterisation of CBP/p300, however, the use of E1A mutants had established that the interaction of E1A with these proteins was required for the induction of DNA synthesis (S-phase entry) in BRK cells, although S-phase could also be induced by a redundant pathway via interaction with pRb (Howe *et al*, 1990). Early region 1A's capacity to induce mitosis required interaction with both pRb and CBP/p300 (Howe and Bayley, 1992). The interaction of E1A with CBP/p300 was also found to be necessary for E1A to transform primary rodent cells in tissue culture, as E1A mutants lacking the capacity to bind CBP/p300 were found to be transformation-defective (Gallimore and Turnell, 2001; Frisch and Mymryk, 2002). Surprisingly, little is known about the mechanism by which E1A promotes cellular transformation through its interaction with CBP/p300. To gain insight into this activity, we must consider the properties of these two proteins. Cyclic AMP response element-binding protein and p300 are two large, highly related lysine acetyltransferases that function as transcriptional coactivators for many sequence-specific transcription factors through the modification of chromatin (Goodman and Smolik, 2000; Iyer *et al*, 2004). The ability of CBP/p300 to activate transcription resides in its capacity to acetylate the core histone proteins associated with enhancer/promoter regions of the genes it activates. This induces conformation changes in chromatin and allows the recruitment of auxiliary proteins to activated promoters. Cyclic AMP response element-binding protein/p300 also have the capacity to acetylate and regulate the activity of a variety of transcription factors including p53, NF-*κ*B and c-Myc. Acetylation in this context affects both the ability of transcription factors to bind DNA, and also to recruit other binding proteins (Goodman and Smolik, 2000). *In vitro* models suggest that sequestration of CBP/p300 by E1A has the general effect of repressing transcription by any factor that utilises these acetyltransferases (Gallimore and Turnell, 2001). However, whether E1A functions *in vivo* to specifically repress CBP/p300 function during tumorigenesis is unknown. It is possible that E1A could utilise CBP/p300 acetyltransferases during tumorigenesis to promote an altered programme of gene expression. Indeed, E1A residue K239 is a major target for CBP/p300 acetylation *in vivo*, and E1A associates with ‘active’ CBP/p300 acetyltransferases from transformed cells (AS Turnell, unpublished data). Acetylation of E1A has been proposed to affect its interaction with the corepressor CtBP, and alter its nuclear localisation by disrupting E1A association with importin-*α* (Zhang *et al*, 2000; Madison *et al*, 2002). It is not yet clear, however, whether acetylation of E1A is required for transformation with either Ras or E1B.

Consistent with the notion that acetyltransferase activity might be required for E1A-mediated transformation, a model has recently been proposed, which suggests that the ability of E1A to induce S-phase depends upon its ability to specifically modulate histone methylation/acetylation status (Ghosh and Harter, 2003). In this case, ‘Tet-on’-inducible expression of E1A in G_0_ initially remodels chromatin by facilitating the demethylation of K9 of histone H3 and the release of repressor E2Fs, and subsequently promotes transcriptional activation and hence G_1_–S progression by promoting acetylation of K9 of histone H3 and the recruitment of activator E2Fs (Ghosh and Harter, 2003). How this is achieved mechanistically however, and the role played by specific demethylases and acetylases in this instance requires further investigation.

Cyclic AMP response element-binding protein/p300 also possess inherent E4 ubiquitin ligase activity, and kinetically enhance the Mdm2-mediated ubiquitylation of p53 (Grossman *et al*, 2003). Whether CBP/p300 facilitates the ubiquitylation of E1A, or whether E1A regulates E4 ligase activity during transformation has not been formally addressed. Interestingly, biallelic mutations in CBP or p300 have been identified in certain human epithelial cancers and reintroduction of wild-type p300 into at least some of these cells suppresses growth (Iyer *et al*, 2004). Cyclic AMP response element-binding protein haploinsufficiency in humans results in Rubinstein–Taybi syndrome, which is not only typified by mental retardation and physical deformities, but also an increased incidence of some types of cancer. Based on these criteria, CBP and p300 exhibit many of the hallmarks of classical tumour suppressor genes (Goodman and Smolik, 2000; Iyer *et al*, 2004).

## FUNCTION OF THE ANAPHASE-PROMOTING COMPLEX/CYCLOSOME (APC/C) AND POTENTIAL ROLE IN TUMORIGENESIS

The APC/C is a macromolecular E3 ubiquitin ligase that coordinates the progression of eukaryotic cells through mitosis, by the timely and selective targeting of protein substrates for 26S proteasome-mediated degradation through ubiquitylation (Harper *et al*, 2002; Peters, 2002). Anaphase-promoting complex/cyclosome selectivity and activity is regulated in a spatially and temporally coordinated manner through the recruitment of Cdc20 or Cdh1, two closely related activators, to distinct phosphorylated-APC/C species. The APC/C targets proteins for destruction through the recognition of one or more loosely defined sequence motifs in the protein substrate: the destruction (D) box, the KEN box or the Aurora A-like (A) box (Harper *et al*, 2002; Peters, 2002). For example, the protein levels of cyclin A and cyclin B1 are regulated in a cell-cycle-specific manner by 26S proteasome-mediated destruction dependent on such APC/C-targeting motifs.

As its name suggests, a primary function of the APC/C is to promote sister-chromatid separation at the metaphase to anaphase transition during mitosis. This is achieved mechanistically through the targeted ubiquitylation and subsequent destruction of the Separase inhibitor, Securin. Upon Securin degradation, Separase cleaves the Cohesin complex component SCC1, which holds sister chromatids together, allowing chromosome partitioning via the mitotic spindle apparatus. The subsequent APC/C-mediated degradation of cyclin B1 allows for mitotic exit and re-entry into G_1_ (Harper *et al*, 2002; Peters, 2002).

Given the key role of the APC/C in coordinating mitosis, and ensuring the fidelity of sister-chromatid separation, it might be anticipated that de-regulation of APC/C function through mutation may lead to genomic instability, the generation of aneuploid daughter cells and cancer. Thus, the APC/C, components thereof, or APC/C regulator proteins might possess inherent tumour suppressor activity to protect against such events. Evidence that individual APC/C components are mutated or deleted in human cancers is scarce. One report suggests that there is a significant loss of APC7 protein expression in ductal breast carcinomas displaying malignant characteristics (Park *et al*, 2005), although how this loss of APC7 affects the function of the holoenzyme is unclear. In addition, analysis of *APC/C* genes from colorectal cancer cell lines suggests that *APC4*, *APC6* and *APC8* are susceptible to mutation, whereas the same study identified one colon tumour from a cohort of 22 (Duke stage not specified) with a mutation in *APC8* (Wang *et al*, 2003). It is obvious that more extensive analyses using tumour material is needed to establish if the APC/C is targeted directly during human tumorigenesis.

Perhaps, a more accurate picture of whether APC/C function is de-regulated during tumorigenesis is a consideration of how aberrant expression of APC/C substrates and APC/C regulators affects cell-cycle progression and ploidy status. In this regard, the overexpression of a number of APC/C substrates can promote genomic instability. For instance, overexpression of the APC/C substrate Securin in mammalian cells promotes cellular transformation through aneuploidy, and moreover, is found specifically overexpressed in a number of human tumours (McCabe and Heaney, 2003). Similarly, overexpression of the APC/C substrate Skp2, an integral component of the SCF ubiquitin ligases that coordinate S-phase entry through the timely destruction of the p21^CIP1/WAF1^ and p27^KIP1^ cyclin-dependent kinase inhibitors, can promote untimely entry into the S-phase and cell-cycle progression; Skp2 overexpression is observed in a number of human cancers with poor prognosis (Nakayama and Nakayama, 2005). Stabilisation of APC/C inhibitors such as Emi1 can also result in genetic instability, through interference with the centrosome duplication cycle (Guardavaccaro *et al*, 2003; Margottin-Goguet *et al*, 2003). Similar arguments for other APC/C substrates and regulators could also be made, but it is apparent from these examples that the de-regulation of the APC/C might play an important role in promoting genomic instability and consequently tumorigenesis.

## RELATIONSHIP BETWEEN CBP/P300 AND THE APC/C

**Figure 2:**
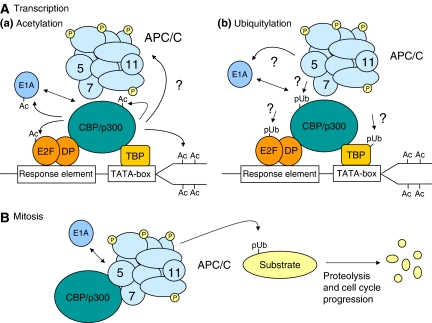
Role of APC/C–CBP/p300 complexes in transcription and cell-cycle control. (**A**(a)) Transcription and acetylation. Cyclic AMP response element-binding protein/p300 function as transcriptional coactivators for sequence-specific DNA-binding transcription factors. These enzymes acetylate histones to alter chromatin accessibility. Cyclic AMP response element-binding protein/p300 also acetylate transcription factors to regulate their activity. Interaction with E1A or the APC/C potentially interferes and/or retargets this activity and E1A is itself a substrate for acetylation. Anaphase-promoting complex5 and/or APC7, as components of the APC/C holoenzyme, interact with CBP/p300 to stimulate inherent CBP/p300 acetyltransferase activity, and CBP/p300-dependent transcriptional activity. Early region 1A might disrupt or mimic APC/C function in this regard through its interaction with CBP/p300. (**A**(b)) Transcription and ubiquitylation. The recruitment of the APC/C to target promoters could potentially regulate CBP/p300 function by promoting CBP/p300 ubiquitylation. The ubiquitylation of the CBP/p300 in this instance could directly affect CBP/p300 acetyltransferase activity, and/or affect CBP/p300 interaction with other proteins, and/or promote proteasomal-mediated degradation of CBP/p300. Early region 1A could interfere with APC/C function in this regard by binding directly to CBP/p300. (**B**) Mitosis. The APC/C complex ubiquitylates cell-cycle regulatory proteins and targets them for proteasomal degradation. Cyclic AMP response element-binding protein functions as an E4 ligase in this regard. Whether acetylation of the APC/C by CBP/p300 regulates APC/C E3 ligase activity in this regard requires further investigation. We propose that E1A might regulate mitotic progression and/or promote genomic instability through interfering directly with APC/C function in mitosis through its ability to bind CBP/p300. Ac, acetylated-residues; pUb, polyubiquitylation; 5, 7 and 11 refer to APC/C subunits. APC11 is the functional ubiquitin ligase. P, phosphorylated residues.

Interestingly, our studies of cellular transformation by E1A identified a previously unrecognised functional link between the APC/C and CBP/p300 (Turnell *et al*, 2005). Specifically, we found that the APC/C components, APC5 and APC7, possess within their primary sequence *bona fide* CBP/p300 protein–protein interaction domains that are homologous to E1A (see Figure 1). Significantly, we determined that both APC5 and APC7, in isolation and as components of the APC/C holoenzyme, stimulated p300 acetyltransferase activity, and hence the transcriptional activity of p300, by enhancing autoacetylation of p300-K1499 in an ubiquitylation-independent manner. The ability of these APC/C subunits to regulate CBP/p300 transcriptional activity suggests that the APC/C might also regulate progression of cells through the G_1_-phase of the cell cycle by coordinating CBP/p300-dependent gene expression programmes that prevent, or allow entry into the S-phase (Figure 2). In this context, the APC/C might also regulate CBP/p300-dependent differentiation pathways. Significantly, we established a role for the APC/C in G_1_ arrest by determining that knockdown of APC5 or APC7 by RNA interference (RNAi) affected the ability of CBP/p300 to potentiate *p21*^*CIP1/WAF1*^ expression in primary human fibroblasts exposed to ionising radiation; the APC/C associates directly with the *p21*^*CIP1/WAF1*^ promoter region. We also established a potential role for the APC/C in the S-phase entry by determining that both APC5 and APC7 can potentiate the CBP/p300-dependent activation of the transcription factor E2F-1; the APC/C associates directly with the E2F-1-regulated gene promoter *CDC6*. Given these findings, it appears that the function of the APC/C is not only important in driving mitosis but also in progression of cells through G_1_ into the S-phase (Figure 2). As the APC/C is a key regulator of mitotic progression, we also investigated potential roles for CBP/p300 in mitosis. Through the use of selective RNAi treatment, we determined that CBP functions to regulate mitotic exit (Figure 2). In this regard, it is our belief that inherent CBP E4 activity enhances APC/C-directed E3 ubiquitylation of substrates such as cyclin B1, through binding the APC/C via APC5 and/or APC7 (Turnell *et al*, 2005).

## E1A PROMOTES CELLULAR TRANSFORMATION BY TARGETING CBP/P300–APC/C COMPLEXES

Given the high degree of cooperation between the APC/C and CBP/p300 in regulating transcription and cell-cycle progression, we investigated whether E1A promoted cellular transformation by ‘targeting’ APC/C–CBP/p300 complexes during the transformation process (Turnell *et al*, 2005). We showed that exogenous expression of wild-type APC5 or APC7 suppressed the ability of E1A to cooperate with E1B or activated-Ras in the transformation of primary rat embryo fibroblasts, whereas the expression of APC5 or APC7 mutants unable to bind CBP/p300 did not suppress E1A-induced transformation. These data clearly established CBP/p300 as a major E1A ‘target’ during transformation. Moreover, in agreement with our earlier proposal, they suggested that APC5 and APC7 might possess inherent tumour suppressor activity. To address this possibility directly, we utilised the R2G E1A mutant that does not bind CBP/p300, and which under normal circumstances is transformation defective. We hypothesised that if wild-type E1A targeted APC/C function directly during transformation through binding CBP/p300, then it might be possible to restore transformation potential to a transformation-defective E1A species, such as R2G E1A, by interfering with normal APC/C function through the selective knockdown of APC5 and/or APC7 protein levels by RNAi. In agreement with our hypothesis, the transformation capacity of R2G E1A, but not wild-type E1A, was increased upon knockdown of *APC5* and/or *APC7* gene expression in primary rat embryo fibroblasts. These studies clearly demonstrated that E1A targets CBP/p300 during transformation to regulate specifically the APC/C.

In light of these observations, we propose that E1A-mediated perturbation/re-orchestration of the normal function of CBP/p300–APC/C complexes is a key event in the induction of cellular transformation (Turnell *et al*, 2005). We suggest that E1A regulates the ability of CBP/p300–APC/C complexes to induce S-phase entry, and promote mitosis. Mechanistically, E1A may induce cellular DNA synthesis by inhibiting the CBP/p300–APC/C-dependent transcriptional activation of genes involved in G_1_ arrest (e.g. *p21*^*CIP1/WAF1*^), and utilising distinct CBP/p300 complexes, potentially in the absence of the APC/C, to induce genes that promote progression into the S-phase. Early region 1A interaction with CBP would also de-regulate the highly coordinated, spatial and temporal activities of the APC/C during mitosis. Here, we suggest that de-regulation of the APC/C by E1A would lead to aberrant mitoses through the direct modulation of CBP-APC/C ubiquitin ligase activity, and potentially by affecting APC/C responses to spindle checkpoint programmes. In consideration of E1A's capacity to target CBP/p300–APC/C complexes during the transformation process, it is conceivable that E1A could also, in a spatially and/or temporally coordinated manner, selectively disrupt APC5–CBP/p300 and/or APC7–CBP/p300 complexes in the context of the APC/C holoenzyme to inhibit some APC/C–CBP/p300 complexes, yet allow for the specific ‘activation’ of others.

A role for E1A in promoting genomic instability is well established. Early studies with HAdV determined that infection of permissive and nonpermissive cells caused both random and nonrandom host cell chromosome aberrations. Aberrant mitoses were also observed in Ad-infected rodent cells, and Ad-induced tumorigenesis was found to be associated with both aneuploidy and polyploidy (see Murray *et al*, 1982 and references therein). Significantly, HAdV-induced genomic instability is predominantly due to E1A expression (Caporossi and Bacchetti, 1990). More recent studies suggest that E1A can induce genomic instability, in part, through its ability to bind Ran, interfere with the centrosome duplication cycle and promote centrosome amplification (De Luca *et al*, 2003). Whether E1A can promote genomic instability through the de-regulation of the APC/C awaits further investigation.

## FUTURE PERSPECTIVES

Although it is apparent that the functions of CBP/p300 and the APC/C are intimately linked, there are many questions regarding their function in transcription and cell-cycle progression that remain to be addressed. Given the enzymatic properties of CBP/p300 and the APC/C, it will be important to define molecular crosstalk between the two complexes. Specifically, does CBP/p300 regulate APC/C function through acetylation of APC/C components, or APC/C regulators? Similarly, as the APC/C is implicated in controlling the protein levels of CBP/p300, it will be important to establish whether the APC/C-directed ubiquitylation of CBP/p300 modulates their ability to function as coactivators in transcription. In this regard, it would be tempting to speculate that in a temporally coordinated manner, the APC/C regulates CBP/p300 function initially through modulation of acetyltransferase activity by direct interaction, and subsequently ubiquitylates CBP/p300 in order to promote other CBP/p300 functions and/or to regulate its proteasomal-mediated destruction. Moreover, as CBP/p300 acetyltransferases and the APC/C E3 ubiquitin ligase both specifically target lysine residues, it will be of particular importance to establish whether there is a cell-cycle-regulated hierarchical sequence of acetylation and ubiquitylation events that determine their function.

In addition, there are other levels of complexity that need to be examined. Particularly, why do both CBP and p300 interact with the APC/C? Presumably, CBP–APC/C complexes and p300–APC/C complexes perform distinct functions during the cell cycle. Indeed, given that it is CBP that functions solely with the APC/C in mitosis, it is unlikely that these proteins operate redundantly. Moreover, why should CBP and p300 bind independently to two different APC/C subunits? Presumably, this reflects the differential activities of APC5 and APC7 in transcription and cell-cycle control and might suggest the existence of discrete subpopulations of APC/C performing distinct functions. Alternatively, this might suggest the existence of APC/C subcomplexes with distinct subunit composition. Discerning the potential differential abilities of E1A to modulate these activities and hence functions will be fundamental in establishing how E1A promotes cellular transformation.
